# (4-Bromo-2-{[2-(morpholin-4-yl)ethyl­imino]­meth­yl}phenolato)dioxido­vanadium(V)

**DOI:** 10.1107/S1600536811035240

**Published:** 2011-09-14

**Authors:** Chen-Yi Wang, Xiang Wu, Feng Cao, Cai-Jun Yuan

**Affiliations:** a Department of Chemistry, Huzhou University, Huzhou 313000, People’s Republic of China

## Abstract

In the title mononuclear dioxidovanadium(V) complex, [V(C_13_H_16_BrN_2_O_2_)O_2_], the V^V^ atom is five-coordinated by one phenolate O, one imine N and one morpholine N atom of the Schiff base ligand, and by two oxide O atoms, forming a distorted square-pyramidal geometry. In the crystal, weak C—H⋯O inter­actions and a short Br⋯Br contact [3.4597 (12) Å] are observed.

## Related literature

For related Schiff base complexes that we have reported recently, see: Wang (2009 ▶, 2011 ▶); Wang & Ye (2011 ▶). For similar oxidovanadium(V) complexes, see: Xie *et al.* (2004 ▶); Gao *et al.* (2005 ▶); Hartung *et al.* (2007 ▶); Romanowski *et al.* (2009 ▶).
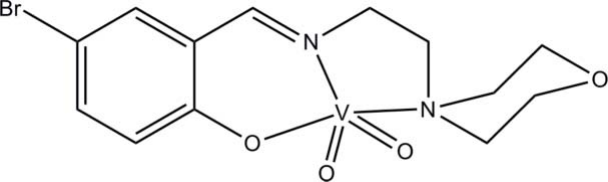


## Experimental

### 

#### Crystal data

[V(C_13_H_16_BrN_2_O_2_)O_2_]
*M* *_r_* = 395.13
Monoclinic, 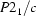

*a* = 21.372 (3) Å
*b* = 6.0892 (15) Å
*c* = 11.372 (3) Å
β = 97.248 (2)°
*V* = 1468.0 (5) Å^3^
*Z* = 4
Mo *K*α radiation
μ = 3.41 mm^−1^
*T* = 298 K
0.17 × 0.13 × 0.13 mm

#### Data collection

Bruker SMART CCD area-detector diffractometer
Absorption correction: multi-scan (*SADABS*; Sheldrick, 1996 ▶) *T*_min_ = 0.595, *T*_max_ = 0.665
11267 measured reflections
3204 independent reflections
2058 reflections with *I* > 2σ(*I*)
*R*_int_ = 0.051

#### Refinement

*R*[*F* ^2^ > 2σ(*F* ^2^)] = 0.044
*wR*(*F* ^2^) = 0.096
*S* = 1.02
3204 reflections
190 parameters
H-atom parameters constrained
Δρ_max_ = 0.61 e Å^−3^
Δρ_min_ = −0.62 e Å^−3^

### 

Data collection: *SMART* (Bruker, 1998 ▶); cell refinement: *SAINT* (Bruker, 1998 ▶); data reduction: *SAINT*; program(s) used to solve structure: *SHELXS97* (Sheldrick, 2008 ▶); program(s) used to refine structure: *SHELXL97* (Sheldrick, 2008 ▶); molecular graphics: *SHELXTL* (Sheldrick, 2008 ▶); software used to prepare material for publication: *SHELXTL*.

## Supplementary Material

Crystal structure: contains datablock(s) global, I. DOI: 10.1107/S1600536811035240/is2767sup1.cif

Structure factors: contains datablock(s) I. DOI: 10.1107/S1600536811035240/is2767Isup2.hkl

Additional supplementary materials:  crystallographic information; 3D view; checkCIF report

## Figures and Tables

**Table 1:** Selected bond lengths (Å)

V1—O4	1.611 (2)
V1—O3	1.622 (3)
V1—O1	1.907 (3)
V1—N1	2.142 (3)
V1—N2	2.159 (3)

**Table 2:** Hydrogen-bond geometry (Å, °)

*D*—H⋯*A*	*D*—H	H⋯*A*	*D*⋯*A*	*D*—H⋯*A*
C7—H7⋯O1^i^	0.93	2.53	3.241 (4)	133
C11—H11*A*⋯O2^ii^	0.97	2.57	3.479 (5)	156

Symmetry codes: (i) 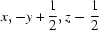; (ii) 
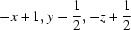
.

**Fig. 1.**
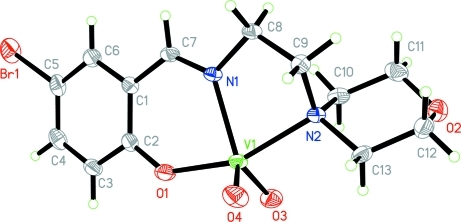
The molecular structure of the title compound, showing the atom-numbering scheme. Displacement ellipsoids are drawn at the 30% probability level.

## supplementary crystallographic information

### Comment

As part of our investigations into new Schiff base complexes (Wang & Ye, 2011; Wang, 2009, 2011), we have synthesized the title compound, a new mononuclear dioxovanadium(V) complex (Fig. 1). The V^V^ atom in the complex is five-coordinated by one phenolate O, one imine N and one morpholine N atom of the Schiff base ligand, and by two oxo O atoms, forming a distorted square pyramidal coordination. The V–O and V–N bond lengths (Table 1) are typical and are comparable with those observed in other similar oxovanadium(V) complexes with Schiff bases (Xie *et al.*, 2004; Gao *et al.*, 2005; Hartung *et al.*, 2007; Romanowski *et al.*, 2009).

### Experimental

5-Bromosalicylaldehyde (1.0 mmol, 0.201 g), 2-morpholin-4-ylethylamine (1.0 mmol, 0.130 g) and VO(acac)_2_ (1.0 mmol, 0.265 g) were dissolved in MeOH (30 ml). The mixture was stirred at room temperature for 10 min to give a yellow solution. After keeping the solution in air for a week, yellow block-shaped crystals were formed at the bottom of the vessel.

### Refinement

H atoms were placed in geometrically idealized positions and constrained to ride on their parent atoms, with C—H distances in the range 0.93–0.97 Å, and with *U*_iso_(H) set at 1.2*U*_eq_(C).

### Crystal data

**Table:**

[V(C_13_H_16_BrN_2_O_2_)O_2_]	*F*(000) = 792
*M**_r_* = 395.13	*D*_x_ = 1.788 Mg m^−^^3^
Monoclinic, *P*2_1_/*c*	Mo *K*α radiation, λ = 0.71073 Å
Hall symbol: -P 2ybc	Cell parameters from 2133 reflections
*a* = 21.372 (3) Å	θ = 2.7–25.1°
*b* = 6.0892 (15) Å	µ = 3.41 mm^−^^1^
*c* = 11.372 (3) Å	*T* = 298 K
β = 97.248 (2)°	Block, yellow
*V* = 1468.0 (5) Å^3^	0.17 × 0.13 × 0.13 mm
*Z* = 4	

### Data collection

**Table:**

Bruker SMART CCD area-detector diffractometer	3204 independent reflections
Radiation source: fine-focus sealed tube	2058 reflections with *I* > 2σ(*I*)
graphite	*R*_int_ = 0.051
ω scans	θ_max_ = 27.0°, θ_min_ = 2.9°
Absorption correction: multi-scan (*SADABS*; Sheldrick, 1996)	*h* = −27→27
*T*_min_ = 0.595, *T*_max_ = 0.665	*k* = −7→7
11267 measured reflections	*l* = −14→14

### Refinement

**Table:**

Refinement on *F*^2^	Primary atom site location: structure-invariant direct methods
Least-squares matrix: full	Secondary atom site location: difference Fourier map
*R*[*F*^2^ > 2σ(*F*^2^)] = 0.044	Hydrogen site location: inferred from neighbouring sites
*wR*(*F*^2^) = 0.096	H-atom parameters constrained
*S* = 1.02	*w* = 1/[σ^2^(*F*_o_^2^) + (0.0418*P*)^2^] where *P* = (*F*_o_^2^ + 2*F*_c_^2^)/3
3204 reflections	(Δ/σ)_max_ < 0.001
190 parameters	Δρ_max_ = 0.61 e Å^−^^3^
0 restraints	Δρ_min_ = −0.62 e Å^−^^3^

### Special details

**Table:**

Geometry. All e.s.d.'s (except the e.s.d. in the dihedral angle between two l.s. planes) are estimated using the full covariance matrix. The cell e.s.d.'s are taken into account individually in the estimation of e.s.d.'s in distances, angles and torsion angles; correlations between e.s.d.'s in cell parameters are only used when they are defined by crystal symmetry. An approximate (isotropic) treatment of cell e.s.d.'s is used for estimating e.s.d.'s involving l.s. planes.
Refinement. Refinement of *F*^2^ against ALL reflections. The weighted *R*-factor *wR* and goodness of fit *S* are based on *F*^2^, conventional *R*-factors *R* are based on *F*, with *F* set to zero for negative *F*^2^. The threshold expression of *F*^2^ > σ(*F*^2^) is used only for calculating *R*-factors(gt) *etc*. and is not relevant to the choice of reflections for refinement. *R*-factors based on *F*^2^ are statistically about twice as large as those based on *F*, and *R*- factors based on ALL data will be even larger.

### Fractional atomic coordinates and isotropic or equivalent isotropic displacement parameters (Å^2^)

**Table:**

	*x*	*y*	*z*	*U*_iso_*/*U*_eq_	
V1	0.28158 (3)	0.31216 (10)	0.45502 (5)	0.02930 (17)	
Br1	0.01304 (2)	−0.30291 (9)	0.10991 (4)	0.06124 (19)	
N1	0.23304 (13)	0.4015 (4)	0.2854 (2)	0.0255 (6)	
N2	0.34664 (13)	0.5238 (4)	0.3783 (2)	0.0253 (7)	
O1	0.21270 (13)	0.1134 (5)	0.4562 (2)	0.0482 (7)	
O2	0.47871 (12)	0.5987 (5)	0.3519 (3)	0.0527 (8)	
O3	0.33864 (12)	0.1419 (4)	0.4953 (2)	0.0442 (7)	
O4	0.27296 (13)	0.4752 (4)	0.5634 (2)	0.0473 (7)	
C1	0.15474 (16)	0.1138 (6)	0.2635 (3)	0.0290 (8)	
C2	0.17008 (17)	0.0235 (6)	0.3773 (3)	0.0329 (9)	
C3	0.13930 (18)	−0.1665 (6)	0.4067 (3)	0.0407 (10)	
H3	0.1501	−0.2298	0.4809	0.049*	
C4	0.09347 (19)	−0.2617 (6)	0.3282 (4)	0.0431 (10)	
H4	0.0730	−0.3876	0.3496	0.052*	
C5	0.07759 (18)	−0.1697 (7)	0.2164 (3)	0.0401 (10)	
C6	0.10726 (17)	0.0147 (6)	0.1835 (3)	0.0372 (9)	
H6	0.0962	0.0748	0.1085	0.045*	
C7	0.18586 (17)	0.3060 (6)	0.2271 (3)	0.0307 (8)	
H7	0.1702	0.3667	0.1542	0.037*	
C8	0.25689 (16)	0.5992 (6)	0.2292 (3)	0.0301 (8)	
H8A	0.2228	0.7031	0.2088	0.036*	
H8B	0.2741	0.5587	0.1573	0.036*	
C9	0.30743 (16)	0.7010 (5)	0.3164 (3)	0.0304 (8)	
H9A	0.3337	0.7968	0.2751	0.036*	
H9B	0.2883	0.7882	0.3737	0.036*	
C10	0.38229 (17)	0.3950 (6)	0.2964 (3)	0.0351 (9)	
H10A	0.3534	0.3487	0.2283	0.042*	
H10B	0.3994	0.2640	0.3371	0.042*	
C11	0.43528 (19)	0.5240 (7)	0.2543 (3)	0.0494 (12)	
H11A	0.4573	0.4323	0.2031	0.059*	
H11B	0.4181	0.6492	0.2084	0.059*	
C12	0.44707 (19)	0.7346 (7)	0.4274 (4)	0.0459 (11)	
H12A	0.4307	0.8634	0.3837	0.055*	
H12B	0.4770	0.7837	0.4934	0.055*	
C13	0.39376 (17)	0.6159 (6)	0.4741 (3)	0.0362 (9)	
H13A	0.4109	0.4971	0.5252	0.043*	
H13B	0.3725	0.7166	0.5219	0.043*	

### Atomic displacement parameters (Å^2^)

**Table:**

	*U*^11^	*U*^22^	*U*^33^	*U*^12^	*U*^13^	*U*^23^
V1	0.0394 (4)	0.0279 (4)	0.0198 (3)	−0.0050 (3)	0.0006 (3)	0.0003 (3)
Br1	0.0583 (3)	0.0709 (4)	0.0544 (3)	−0.0337 (3)	0.0067 (2)	−0.0207 (2)
N1	0.0354 (17)	0.0205 (16)	0.0202 (15)	−0.0021 (13)	0.0015 (13)	0.0013 (12)
N2	0.0338 (17)	0.0198 (16)	0.0215 (14)	−0.0002 (13)	0.0006 (12)	−0.0009 (12)
O1	0.0541 (18)	0.0570 (19)	0.0305 (15)	−0.0253 (15)	−0.0059 (13)	0.0126 (14)
O2	0.0331 (16)	0.068 (2)	0.0572 (18)	−0.0001 (15)	0.0048 (14)	0.0167 (17)
O3	0.0503 (17)	0.0344 (16)	0.0459 (16)	0.0026 (13)	−0.0017 (13)	0.0139 (13)
O4	0.0660 (19)	0.0461 (18)	0.0318 (14)	−0.0093 (15)	0.0135 (13)	−0.0113 (13)
C1	0.034 (2)	0.025 (2)	0.029 (2)	−0.0045 (16)	0.0059 (16)	−0.0027 (16)
C2	0.033 (2)	0.034 (2)	0.033 (2)	−0.0049 (18)	0.0085 (17)	−0.0014 (17)
C3	0.045 (2)	0.045 (3)	0.033 (2)	−0.011 (2)	0.0064 (18)	0.0057 (19)
C4	0.048 (3)	0.033 (2)	0.051 (3)	−0.0123 (19)	0.018 (2)	−0.003 (2)
C5	0.037 (2)	0.045 (3)	0.039 (2)	−0.0175 (19)	0.0094 (18)	−0.019 (2)
C6	0.039 (2)	0.041 (3)	0.031 (2)	−0.0065 (19)	0.0039 (17)	−0.0050 (18)
C7	0.037 (2)	0.037 (2)	0.0181 (17)	0.0017 (18)	0.0026 (15)	−0.0014 (17)
C8	0.037 (2)	0.024 (2)	0.0287 (19)	0.0028 (16)	0.0010 (16)	0.0062 (16)
C9	0.033 (2)	0.022 (2)	0.035 (2)	0.0019 (16)	0.0016 (16)	−0.0003 (17)
C10	0.045 (2)	0.030 (2)	0.030 (2)	0.0120 (18)	0.0030 (17)	0.0002 (17)
C11	0.047 (3)	0.065 (3)	0.039 (2)	0.011 (2)	0.017 (2)	0.010 (2)
C12	0.041 (2)	0.043 (3)	0.051 (3)	−0.008 (2)	−0.008 (2)	0.007 (2)
C13	0.041 (2)	0.038 (2)	0.027 (2)	−0.0040 (19)	−0.0072 (17)	−0.0010 (17)

### Geometric parameters (Å, °)

**Table:**

V1—O4	1.611 (2)	C4—C5	1.392 (5)
V1—O3	1.622 (3)	C4—H4	0.9300
V1—O1	1.907 (3)	C5—C6	1.365 (5)
V1—N1	2.142 (3)	C6—H6	0.9300
V1—N2	2.159 (3)	C7—H7	0.9300
Br1—C5	1.899 (4)	C8—C9	1.504 (5)
N1—C7	1.275 (4)	C8—H8A	0.9700
N1—C8	1.483 (4)	C8—H8B	0.9700
N2—C9	1.487 (4)	C9—H9A	0.9700
N2—C13	1.497 (4)	C9—H9B	0.9700
N2—C10	1.497 (4)	C10—C11	1.505 (5)
O1—C2	1.314 (4)	C10—H10A	0.9700
O2—C12	1.423 (5)	C10—H10B	0.9700
O2—C11	1.428 (4)	C11—H11A	0.9700
C1—C2	1.406 (5)	C11—H11B	0.9700
C1—C6	1.410 (5)	C12—C13	1.502 (5)
C1—C7	1.433 (5)	C12—H12A	0.9700
C2—C3	1.392 (5)	C12—H12B	0.9700
C3—C4	1.368 (5)	C13—H13A	0.9700
C3—H3	0.9300	C13—H13B	0.9700
			
O4—V1—O3	109.41 (14)	N1—C7—C1	126.0 (3)
O4—V1—O1	102.89 (13)	N1—C7—H7	117.0
O3—V1—O1	98.38 (13)	C1—C7—H7	117.0
O4—V1—N1	116.30 (13)	N1—C8—C9	107.9 (3)
O3—V1—N1	132.70 (12)	N1—C8—H8A	110.1
O1—V1—N1	83.17 (11)	C9—C8—H8A	110.1
O4—V1—N2	94.75 (12)	N1—C8—H8B	110.1
O3—V1—N2	89.77 (12)	C9—C8—H8B	110.1
O1—V1—N2	156.74 (10)	H8A—C8—H8B	108.4
N1—V1—N2	75.39 (10)	N2—C9—C8	109.1 (3)
C7—N1—C8	116.0 (3)	N2—C9—H9A	109.9
C7—N1—V1	127.9 (2)	C8—C9—H9A	109.9
C8—N1—V1	116.1 (2)	N2—C9—H9B	109.9
C9—N2—C13	111.1 (3)	C8—C9—H9B	109.9
C9—N2—C10	112.7 (3)	H9A—C9—H9B	108.3
C13—N2—C10	107.5 (3)	N2—C10—C11	112.7 (3)
C9—N2—V1	105.7 (2)	N2—C10—H10A	109.0
C13—N2—V1	109.74 (19)	C11—C10—H10A	109.0
C10—N2—V1	110.0 (2)	N2—C10—H10B	109.0
C2—O1—V1	136.7 (2)	C11—C10—H10B	109.0
C12—O2—C11	110.0 (3)	H10A—C10—H10B	107.8
C2—C1—C6	119.5 (3)	O2—C11—C10	111.1 (3)
C2—C1—C7	121.5 (3)	O2—C11—H11A	109.4
C6—C1—C7	119.0 (3)	C10—C11—H11A	109.4
O1—C2—C3	119.3 (3)	O2—C11—H11B	109.4
O1—C2—C1	121.8 (3)	C10—C11—H11B	109.4
C3—C2—C1	118.9 (3)	H11A—C11—H11B	108.0
C4—C3—C2	121.1 (4)	O2—C12—C13	111.9 (3)
C4—C3—H3	119.4	O2—C12—H12A	109.2
C2—C3—H3	119.4	C13—C12—H12A	109.2
C3—C4—C5	119.8 (4)	O2—C12—H12B	109.2
C3—C4—H4	120.1	C13—C12—H12B	109.2
C5—C4—H4	120.1	H12A—C12—H12B	107.9
C6—C5—C4	120.9 (4)	N2—C13—C12	113.1 (3)
C6—C5—Br1	120.1 (3)	N2—C13—H13A	109.0
C4—C5—Br1	119.0 (3)	C12—C13—H13A	109.0
C5—C6—C1	119.7 (4)	N2—C13—H13B	109.0
C5—C6—H6	120.1	C12—C13—H13B	109.0
C1—C6—H6	120.1	H13A—C13—H13B	107.8

### Hydrogen-bond geometry (Å, °)

**Table:**

*D*—H···*A*	*D*—H	H···*A*	*D*···*A*	*D*—H···*A*
C7—H7···O1^i^	0.93	2.53	3.241 (4)	133
C11—H11A···O2^ii^	0.97	2.57	3.479 (5)	156

Symmetry codes: (i) *x*, −*y*+1/2, *z*−1/2; (ii) −*x*+1, *y*−1/2, −*z*+1/2.
